# A blended face-to-face and eHealth lifestyle intervention on physical activity, diet, and health outcomes in Hong Kong community-dwelling older adults: a study protocol for a randomized controlled trial

**DOI:** 10.3389/fpubh.2024.1360037

**Published:** 2024-05-07

**Authors:** Min Yang, Yanping Duan, Sonia Lippke, Wei Liang, Ning Su

**Affiliations:** ^1^Department of Sport, Physical Education and Health, Faculty of Social Sciences, Hong Kong Baptist University, Kowloon, Hong Kong SAR, China; ^2^School of Business, Social and Decision Sciences,Constructor University, Bremen, Germany; ^3^College of Physical Education, Shenzhen University, Shenzhen, China

**Keywords:** physical activity, diet, health outcomes, older adults, blended intervention

## Abstract

**Background:**

Aging individuals are vulnerable to various Noncommunicable Diseases (NCDs). Different behaviors are closely related to a decreased risk of suffering from NCDs: sufficient Physical Activity (PA) (e.g., at least 150 mins Moderate-to-vigorous Physical Activity (MVPA) per week) and a healthy daily diet (e.g., at least five portions of Fruit and Vegetable Intake (FVI), 5–6 taels (189.0–226.8 g) Meat, Fish, Egg and Alternatives (MFEA)). Traditional face-to-face interventions were effective in behavior change. However, it was revealed to be resource-intensive and limited transfer due to poor self-regulation skills outside of face-to-face sessions. Thus, eHealth could be a supplement for older adults outside traditional face-to-face settings. The blended approach combining these two interventions might optimize the intervention effects on lifestyle behavior initiation and maintenance, but little research can be found among Hong Kong older adults. Therefore, the study aims to test a blended intervention to promote PA, diet, and health outcomes among Hong Kong community-dwelling older adults.

**Methods:**

This study will adopt a 10-week three-arm randomized controlled trial. The blended group will receive weekly (1) two 60-min face-to-face sessions with one for PA and one for diet, and (2) two web-based sessions with one for PA and one for diet. The face-to-face group will receive the same intervention content as the face-to-face sessions in the blended group. The control condition will receive a biweekly telephone call. The outcomes will include MVPA (minutes/week), FVI (portions/day), MFEA consumption (taels/day), social-cognitive factors (self-efficacy, planning, social support, action control), physical health outcomes (clinical indicators, senior physical fitness), mental health outcomes (depression, loneliness) and health-related quality of life. Data collection will be implemented at the pre-test, post-test, and 3-month follow-up test.

**Discussion:**

This is the first study evaluating a blended intervention promoting multiple health behaviors among Hong Kong community-dwelling older adults. If the effect of the blended intervention is superior to the traditional face-to-face group and the control group, it will enrich lifestyle intervention approaches and can be applied to older adults, helping them obtain health benefits. Furthermore, a better understanding of mechanisms will also have implications for theory-building.

**Clinical trial registration:**

https://www.isrctn.com/ISRCTN32329348, ISRCTN32329348.

## 1 Introduction

Noncommunicable diseases (NCDs), such as cardiovascular diseases, cancer, diabetes, and obesity, are responsible for the mortality of approximately 41 million individuals annually, accounting for a staggering 74% of all global deaths (1). NCDs are of high prevalence due to a confluence of factors, including the globalization of unhealthy lifestyles and the demographic shift toward an aging population (2). Physical inactivity and unhealthy dietary patterns contribute to the manifestation of metabolic risk factors, such as elevated blood pressure, hyperglycemia, dyslipidemia, and obesity (3, 4). However, over one-quarter of adults were physically inactive, while over 3 million NCD-related deaths per year could be averted by increasing physical activity (PA) (5). The evidence indicates that 28% of adults were physically inactive and did not meet the World Health Organization (WHO) recommendation level of PA (5). The survey conducted in America suggested that the prevalence of physical inactivity significantly increases with age, with 26.9% among those aged 65–74 years and 35.3% among those aged ≥75 years (6). In Hong Kong, 24.8% of adults do not meet the WHO PA recommendation (at least 150 minutes Moderate-to-vigorous Physical Activity (MVPA) per week), while the percentage among older people is higher than that of other age groups (7).

Less than 50% of older adults adhere to the recommended five portions per day of fruit and vegetables (8, 9). An estimated 3.9 million deaths worldwide were attributable to inadequate fruit and vegetable consumption in 2017 (10). For Fruit and Vegetable Intake (FVI), most Hong Kong older adults consume less than five servings per day, in particular, 94.8% in the 65–74 age group, 96.6% in the 75–84 age group, and 99.3% in the 85 or above age group (7). As individual household incomes rise, global meat consumption and average *per capita* meat consumption are also increasing, driven by globalization and evolving food preferences, which heightened meat consumption is closely linked to the development of various diseases (11), particularly NCDs (12). It is essential to avoid excessive consumption of certain foods, such as meat consumption. In Hong Kong, the *per capita* meat consumption was 375 g, which ranked first in the world (13), while older people are recommended to have 5–6 taels Meat, Fish, Egg and Alternatives (MFEA) (189.0–226.8 g) per day (7). Engaging in multiple risk behaviors can lead to adverse health effects. Healthy lifestyle interventions among older adults are essential and meaningful to prevent harmful effects on individual health, medical systems, and medical costs (14).

Face-to-face workshops enable providers to deliver individualized interventions, facilitate interactive communication, and protect privacy (15). Empirical evidence supports the efficacy of face-to-face interventions in promoting health behaviors, such as PA and dietary choices (16, 17). Notwithstanding the practical support of face-to-face health interventions, this approach exhibits several limitations. Previous research also indicated significant barriers to older adults’ attendance and adherence to health behavior change intervention, including lack of perceived social support, low motivation, and poor self-regulation skills (18). The adherence rate of stand-alone face-to-face intervention on health promotion among older adults decreased over time (19).

eHealth is an emerging channel for delivering healthcare services and information through the Internet and associated technologies and media (e.g., computers and smartphones). It can complement face-to-face interventions for older adults in their homes or beyond traditional care settings (20). eHealth efficiently provides personalized feedback and long-term monitoring related to healthy lifestyles, which can enhance the maintenance of a healthy lifestyle over an extended period (21).

This study used the Health Action Process Approach (HAPA) model as a theoretical backdrop for the eHealth intervention (22). The HAPA model posited two phases (motivational and volitional) in the process of behavior change (23). During this dynamic process, individuals move from forming a behavioral intention to executing and sustaining a specific health behavior. In the process of behavioral change, several psychosocial factors play a crucial role. The motivational stage focuses on developing a behavioral intention, with action self-efficacy, outcome expectancies, and risk perceptions identified as contributing factors (24, 25). After individuals have established a behavioral intention, volitional factors such as action planning, coping planning, maintenance and recovery self-efficacies, and external resources like social support become essential in overcoming the intention-behavior gap (26). These factors collectively facilitate the execution and maintenance of the desired behavior (27). The widespread approval of the HAPA model in promoting various health behaviors among adults underscores its applicability (28). The effect of the techniques can be explained by theory, mainly relating to Behavior Change Techniques (27, 29).

However, eHealth intervention may also have disadvantages compared to face-to-face interventions. It demands specific skills, including electronic device functionality and reading comprehension proficiency. Providing individualized attention from professional medical care may pose challenges for caregivers (30). Furthermore, adherence is a crucial challenge in any eHealth intervention program, as many participants drop out before completion (31, 32).

In recent years, the blended intervention combining face-to-face workshops and eHealth intervention anticipates that the strengths of one delivery mode would offset the weaknesses of the other (33–36). A recent meta-analysis reported that the blended intervention can significantly promote total PA levels and diet behavior among adults (37). However, whether a blended intervention is more effective than traditional face-to-face interventions is unclear. Also, only limited findings reported the effectiveness of blended interventions in promoting PA and diet among older adults.

The findings of this study aim to fill the research gap, and its research design will be outlined in the following sections. There are two research objectives in this study. The primary aim is to compare the intervention effects between blended, face-to-face, and control groups in primary outcomes, including PA, diet (FVI and MFEA), and adherence to the health recommendations of PA and diet (FVI and MFEA). The second objective is to compare the intervention effects between blended, face-to-face, and control groups in secondary outcomes, including social-cognitive variables of behavior change (i.e., self-efficacy, planning, social support, and action control), physical health outcomes (i.e., clinical indicators, senior physical fitness), mental health outcomes (i.e., depression, loneliness), and health-related quality of life (HRQoL).

## 2 Methods and design

### 2.1 Study design and procedure

**Figure 1:**
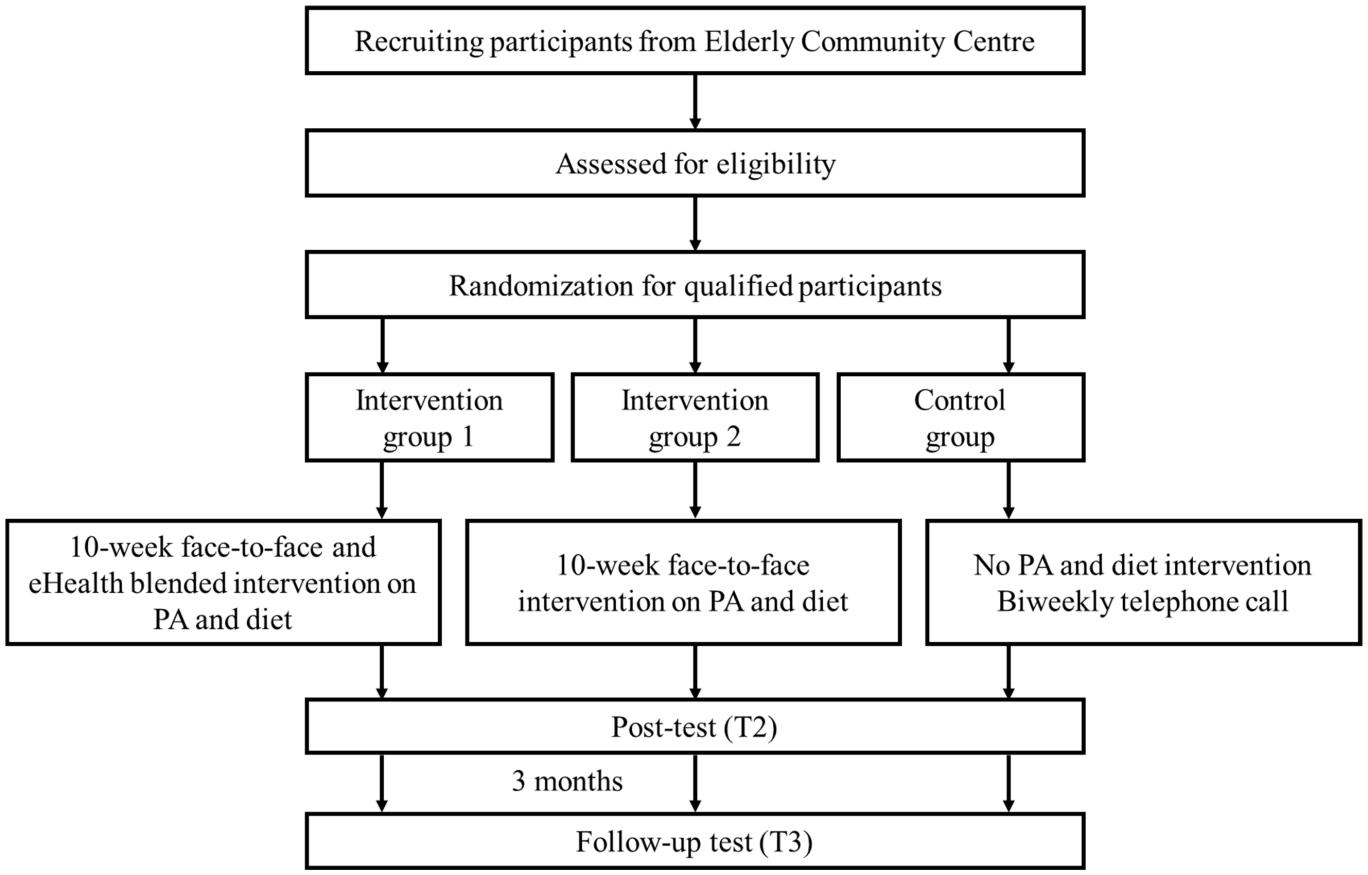
The flowchart of the study.

This study is a single-blind randomized controlled trial (RCT) comprising three groups: a blended face-to-face and eHealth intervention group (IG-1), a stand-alone face-to-face intervention group (IG-2), and a control group (CG). Evaluation will be conducted at pre-intervention (T1), post-intervention (T2), and 3 months follow-up after intervention completion (T3). The target population will be Hong Kong community-dwelling older adults. Participants will be recruited and randomly assigned into one of three groups. The intervention givers (i.e., PA coach and nutritionist) will be blinded from the assignment results to the intervention. The study process is presented in Figure 1.

### 2.2 Target population

Participants will be recruited from two Senior Centers in Hong Kong. The e-poster and video will be advertised by the staff in the Senior Centers. There are over 3,000 members in these two centers. Therefore, recruiting participants by advocating for the Senior Centers is feasible and advantageous. Potential study participants will be informed that there will be a health promotion program in which they can obtain health-related programs and receive a $300HKD (about $39USD) supermarket gift card as an incentive if the participants complete the 10-week health project and finish three times data collection.

Once the older adults express their interests, they will be encouraged to register in the program. After finishing all the registration and eligible screening, they will receive the study consent forms. The eligible criteria include: (1) aged 65 years and above; (2) owning a smartphone and having access to the Internet; (3) passing the Physical Activity Readiness Questionnaire (PAR-Q); (4) having sufficient reading and listening skills in Cantonese; (5) at least one health behavior (PA, FVI, MFEA) fails to meet the recommended health guideline. Exclusion criteria will include: (1) under special situations that seriously affected their diet (e.g., oral allergy syndrome) and mobility (e.g., physical disability); (2) meet all the health guidelines of PA, FVI, and MFEA; (3) participate in other ongoing project related to PA and diet intervention. Then, all participants will be allocated to one of three groups with an equal ratio (in a 1:1:1 ratio). The group allocation will be blinded. The researchers will provide the individual web link for the blended intervention group participants.

The sample size was calculated using G*Power 3.1 software. For achieving a medium effect size (Cohen’s *f* 0.26) on PA and diet based on a previous similar blended study among adults (35), with a statistical power (1-*β*) of 0.8 and an alpha of 0.05 (38), a total of 91 participants is required for the three groups (IG-1, IG-2, and CG). Considering the dropout rate of approximately 20% (35), a total of *N* = 114 participants is required to enable a robust evaluation.

### 2.3 Intervention content

#### 2.3.1 Face-to-face intervention group

Previous findings indicated that the majority of the face-to-face sessions conducted exercise training and education among older adults without theory backdrop (39). The participants in the face-to-face intervention group will engage in two 60-min sessions per week, including one session for PA at the gym and one for diet at the neighborhood senior center (40) for 10 weeks. Each PA session will consist of two parts: health education on PA and health (10 minutes) and physical exercise (50 minutes). Each session will include a warm-up exercise (5 minutes), a main exercise (40 minutes), and a cool-down exercise (5 minutes). The main activity will consist of diverse components such as aerobic training, resistance training, balance training, and mindfulness meditation training. The feedback will be given during PA courses if the participants have questions. Each PA session will be delivered by a PA coach with the assistance of two to four research helpers to ensure safety and feasibility.

Each diet session will consist of two parts: health education on diet (30 minutes) and nutrition counseling (30 minutes). The education topics, such as dietary fiber, cholesterol, fruits and vegetables, and red and white meat, were developed based on suggestions from a nutritionist. The nutrition counseling will include a 15 minutes group discussion with 3–4 participants in one group to design daily dishes and 15 minutes of dish sharing. Personalized nutritional advice will be offered by the nutritionist accordingly. Each diet session will be delivered by a nutritionist with the assistance of one research helper.

#### 2.3.2 Face-to-face and eHealth blended intervention group

In addition to attending all face-to-face sessions, participants in the blended intervention group will participate in a theory-based and web-based behavioral change promotion eHealth intervention, including one program for PA and one program for diet each week. A mobile-based website, namely Perfect Diet and Exercise (in Cantonese: “食” 全十美, 行大 “運”), will be established to facilitate the intervention. The website’s homepage will have the PA module and the diet (FVI and MFEA) module. Participants can access each module to take part in respective online learning activities. Each module will consist of two sections. Section 1 comprises a theory-based 10-week intervention program targeting social-cognitive predictors of PA/diet (FVI and MFEA). Participants will access this section once a week. Section 2 is a data repository platform collecting former exercises and incentive activities. Participants can access their files at any time throughout the 10-week intervention period, including behavior records of PA/diet, action planning of PA/diet, coping planning of PA/diet, and “my diary” about PA/diet behaviors. The weekly eHealth intervention component will be developed based on the Health Action Process Approach (HAPA) (41). Following the HAPA-based social-cognitive variables, the weekly intervention content will be designed accordingly (see Table 1).

**Table 1:** Theory-based intervention variables and behavior change techniques (BCTs) in 10 weeks.

Time	Social-cognitive variables	Behavior change techniques (BCTs)	BCTs related strategies
Week1	Motivational self-efficacy Action planning Social support	1.4 Action planning 2.2 Feedback on behavior 3.2 Social support practical 3.3 Social support emotional 7.1 Prompts/cues 15.1 Verbal persuasion about capability	a) Asking participants about their MVPA/diet behavior and providing personally tailored feedback b) Prompting verbal persuasion about the capability of performing MVPA/diet (targeting motivational self-efficacy) c) Asking participants to keep their plan in mind can help them successfully perform the wanted behavior (targeting Action control) d) Giving examples of successful action plans for MVPA/diet adherence (targeting action planning) e) Asking participants to make specific action plans of when, where, and how to perform MVPA/diet (targeting action planning) f) Asking participants to review social support on MVPA/diet initiation they received and give examples of successful social support for MVPA/diet adherence (targeting perceived social support)
Week 2–4	Motivational self-efficacy Action planning Social support	1.4 Action planning 2.2 Feedback on behavior 3.2 Social support practical 3.3 Social support emotional 7.1 Prompts/cues 15.1 Verbal persuasion about capability	Some strategies used in Week 1 (a, b, c, d, f) will be further applied in Week 2–4 g) Giving examples of successful cases of MVPA/diet participation (further targeting motivational self-efficacy) h) Participants will be asked to access the execution of action plans and adjust the previous action plans if necessary (targeting revision of action planning)
Week 5	Volitional self-efficacy Coping planning Action control Social support	1.2 Problem solving (coping panning) 2.2 Feedback on behavior 2.3 Self-monitoring of behavior 3.2 Social support practical 3.3 Social support emotional 7.1 Prompts/cues 10.1. Material incentive behavior 15.1 Verbal persuasion about capability	Some strategies used in Week 1–4 (a, b, d, f) will be further applied in Week 5 i) Giving examples of successful cases of MVPA/diet maintenance and recovery (targeting VSE) j) Giving examples of successful coping plans for MVPA/diet maintenance and recovery (targeting coping planning) k) Providing guidance (e.g., If-then algorithm) on how to set coping plans to overcome potential barriers when performing MVPA/diet (targeting coping planning) l) Guiding how to write a reflective diary (MVPA/diet behavior performed and emotional experience) to record and self-monitor their daily MVPA/diet (targeting action control)
Week 6–10	Volitional self-efficacy Coping planning Action control Social support	1.2 Problem solving (coping panning) 2.2 Feedback on behavior 2.3 Self-monitoring of behavior 3.2 Social support practical 3.3 Social support emotional 7.1 Prompts/cues 10.1. Material incentive behavior 15.1 Verbal persuasion about capability	Some strategies used in Week 1–5 (a, b, d, f, l) will be further applied in Week 6–10 m) Participants will be asked to review the execution of coping plans and adjust the coping plans in Weeks 6–9 if necessary (targeting revision of coping planning)

**Figure 2:**
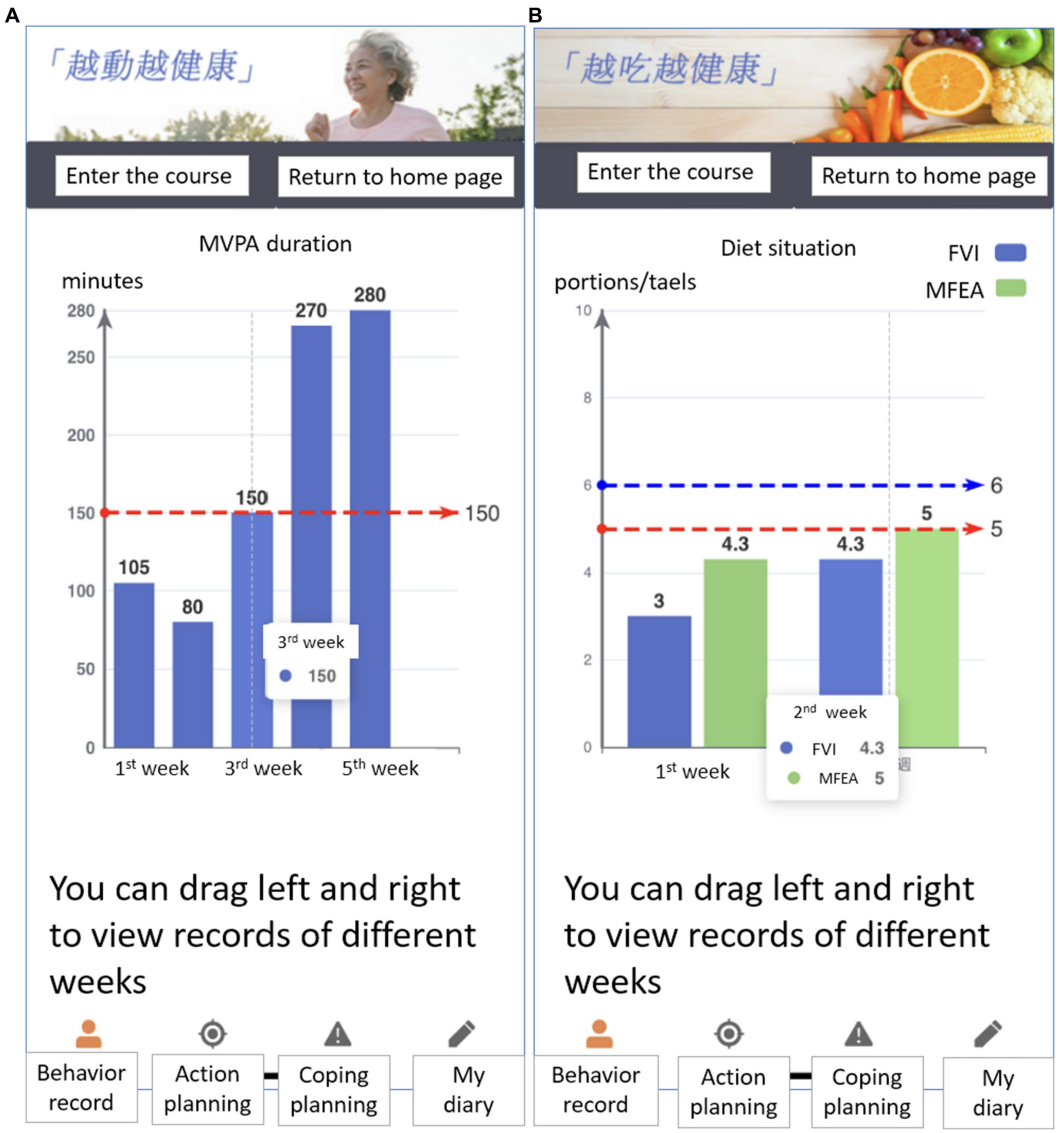
Individualized and normative feedback regarding PA per week during the past 5 weeks **(A)** as well as portions of fruit and vegetable, and taels of meat, fish, egg, and alternatives consumption in average per day during the past 2 weeks **(B)** for the blended intervention group.

In addition, the intervention will apply different behavior change techniques (BCTs) to facilitate behavior (42). The detailed strategies of BCTs are presented in Table 1. For example, the participants will receive two types of feedback in the weekly web-based health session. This will include individualized feedback on their behavior performance in the 1st week, 2nd week, 3rd week, 4th week, and 5th week, respectively. Furthermore, the normative feedback on the criterion-based behavior recommendations (e.g., accumulated at least 150 minutes with moderate intensity of PA per week, five portions of FVI per day, 5–6 taels of MFEA per day) (see Figure 2) will be contained.

Each weekly web-based section for PA and diet will last about 15 minutes. In the first week, once the participants complete the face-to-face intervention, the researcher will immediately organize a web-based intervention course for older adults at the PA gymnasium and the senior center for diet, respectively. The researcher will first introduce the website’s content and then demonstrate the steps to use this web-based program. Afterward, research helpers will guide participants in accessing the website and finishing the online study. In the 2nd week, the web-based intervention course will be continuously offered at the gym and the senior center to ensure older adults are familiar with the website’s usage. From the 3rd week, participants will be encouraged to finish the online courses at home to support transfer.

#### 2.3.3 Control group

The participants in the control group will receive a biweekly telephone call. Each call will last about 5 minutes. The participants will be asked several health-related questions, such as “Has your physical activity changed in the last 2 weeks?” “If it changed, what are the reasons for the increase or decrease?” “Have you changed your diet in the last 2 weeks?” and “What has changed? What are the reasons for the increase or decrease if it changed?”

### 2.4 Outcome evaluation of the intervention

All the outcomes will be measured at T1 (baseline pre-test), T2 (post-test), and T3 (follow-up). Only demographics will be collected at T1. All measurement scales will be in Cantonese, which has been widely used in previous studies conducted in Hong Kong.

#### 2.4.1 Primary outcomes: behavior indicators

##### 2.4.1.1 Physical activity (PA)

The waist-worn accelerometer (wGT3XBT, ActiGraph Inc., Pensacola, FL, USA) will objectively assess moderate-to-vigorous intensity physical activity (MVPA) during the last 7 days. The participants will be guided to wear accelerometers attached to elastic belts around their waists during all waking hours for seven consecutive days. They must remove the waist-worn accelerometers only during sleeping or water-based activities (43). The Troiano wear time validation algorithm will be applied to activity counts (44). With this algorithm, periods of 60+ consecutive minutes of continuous zero counts with a tolerance of up to 2 minutes of activity counts between 0 to 100 counts/min will be classified as non-wear time (44). Data of at least 10 hours per day for at least 3 days will be considered valid and included in data analysis according to a previous systematic review (45). For the estimated PA intensity, the Sasaki et al. cut-point will be applied, 200–2,690 counts/min is for LPA, and ≥ 2,691 counts/min is for MVPA (46).

##### 2.4.1.2 Fruit and vegetable intake (FVI) and meat, fish, egg & alternatives (MFEA)

To assess the FVI and MFEA during the past 7 days, participants will be asked to finish a diet record booklet and fill in how many portions of fruit, vegetable, and fruit and vegetable juice as well as how many taels of meat, fish, egg, and alternatives they have consumed within 1 day. The items will include “On the first day, I ate xxx portions of vegetables, I ate xxx portions of fruits, I drank xxx portions of vegetable and fruit juice. In total, xxx portions fruit and vegetables” and “On the first day, I ate xxx taels of meat (including fish), I ate xxx taels of eggs, I ate xxx taels of alternatives and a total of xxx taels meat, fish, egg, and alternatives.” The average number of FVI and MFEA they ate daily will be calculated based on the records they filled out during the past 7 days. These items are developed based on the diet recommendations provided by the Center for Health Protection Department of Health in Hong Kong (47). One tael is equal to 37.799 grams. Each participant will receive an assessment guide booklet on the portion of fruit and vegetables and the tael of MFEA.

#### 2.4.2 Secondary outcomes: health outcomes

##### 2.4.2.1 Physical health outcomes

The Senior Fitness Test (SFT) Manual will be applied to assess the physical fitness of participants (48). The testing materials have been widely used in many countries and translated into many languages, including Chinese (49). The physical fitness testing includes the measurements of the 30-Second Chair Stand Test (lower body strength), 30-Second Arm Curl Test (upper body strength), 2-Minute Step Test (aerobic endurance), Chair Sit-and-Reach Test (lower body flexibility), Back Scratch Test (upper body flexibility), 8-foot Up-and-Go Test (agility and dynamic balance), and body mass index (BMI). Detailed testing guidance will be provided during testing. For example, 30-Second Chair Stand Test will be used to test lower body strength. Required measuring tools include a straight back or folding chair without armrests (seat 17 inches/43 cm high) and a stopwatch. The participant will be asked to sit in the middle of the seat, with their feet shoulder-width apart, flat on the floor. The arms should be crossed at the wrists and held close to the chest. The participant will be required to keep their back straight and keep their arms against the chest. After listening to the “Go” instruction, the participant should rise to a full standing position, and then sit back down again. The participant should repeat this action for 30 s. If the participant has completed a full stand from the sitting position when the time has elapsed, the final stand is counted in the total. The reliability of SFT items ranged from 0.80 to 0.98 (49).

The blood pressure will be measured by an automated portable device (Omron HEM-7121 Standard, Japan) following the procedure guidelines (50). Participants will be asked to sit quietly for 5–10 minutes before their blood pressure is measured. The blood pressure will then be recorded on an electronic screen and written on paper for recording. The unit would be mmHg. Capillary blood samples will be collected by finger-prick (51). The glycosylated hemoglobin (HbA1c, mmol/mol) and lipid (Total Cholesterol, CHOL; triglycerides, TG; high-density lipoprotein, HDL; mg/dL) will be tested by Point of Care (POC) Cobas b 101 system (Roche Diagnostics, Mannheim, Germany). A 30 uL fingertip blood sample will be collected from participants and immediately tested using two test trips. The system can generate HbA1c and lipid assay results by using 2 uL for the HbA1c test and 19 uL for the lipid test by finger-prick blood samples within 15 minutes (52). Participants will be asked to stay in a fasted state for 12 hours before the blood test.

##### 2.4.2.2 Mental health outcomes

The Geriatric Depression Scale 15-item (GDS-15) - Cantonese Version will be used to assess depression among participants. The validity and reliability of the GDS-15 were validated as satisfactory among Chinese older adults in previous studies and were regarded as a valuable method for measuring depression in older adults (Cronbach’s *α* = 0.81–0.83) (53, 54). Participants will be asked 15 items referring to the past week, such as “Are you satisfied with your life?” and “Have you dropped many of your activities and interests?.” Answers are indicated as yes “1” or no “0.” For items 1, 5, 7, 11, and 13, the answer is yes “0” or no “1.”

The Chinese translation of the 6-item De Jong Gierveld Loneliness Scale will be applied to assessing loneliness. This scale was reliable and valid for measuring loneliness among Hong Kong older adults (Cronbach *α* = 0.76) (55). The participants will be informed, “Please see if these statements describe your situations or feelings now. If you think that they are describing you, answer yes; if you disagree with the statements, answer more or less; if you do not think that they are describing you, answer no.” Participants will be asked six items including two domains (emotional loneliness and social loneliness), such as “I experience a general sense of emptiness” and “There are plenty of people I can rely on when I have problems.” For the first three items related to emotional loneliness, answers are indicated as yes “1,” more or less “1,” and no “0.” For the last three items related to social loneliness, answers are indicated as yes “0,” more or less “1,” and no “1.”

##### 2.4.2.3 Social-cognitive factors

The validated questionnaires will be used to assess the four social-cognitive variables (56–58), including Questionnaires of Self-efficacy (five items for each behavior) (59), Questionnaires of Planning (six items for PA, four items for each diet behavior) (60, 61), Questionnaires of Social Support (3 items for each behavior) (62), and Questionnaires of Action Control (6 items for each behavior) (63).

##### 2.4.2.4 Health-related quality of life (HRQoL)

A brief version of the World Health Organization’s Quality of Life questionnaire (WHOQOL-BREF HK) will be used to evaluate the HRQoL (62) in Hong Kong. Participants will be first asked about their general quality of life using the question “How would you rate your quality of life?” on a two-item five-point scale (1 = very poor; 5 = very good). The physical health subdomain with seven items will also be used (Cronbach’s *α* = 0.71), such as “Are you satisfied with your ability to do everyday things?” and “Do you have enough energy to cope with daily life?” (1 = very dissatisfied; 5 = very satisfied). However, for item 3 and item 4, the participants will be asked, “Do you need medical help to cope with everyday life?” and “Do you feel pain and discomfort holding you back?” (1 = an extreme amount; 5 = not at all).

##### 2.4.2.5 Demographics

Items will include name, mobile number, gender, age, marital status, number of children, educational level, professional status, household income, and history of chronic diseases.

### 2.5 Pilot study

A pilot study of blended intervention was run before the main study. The aim was to ensure that the recruitment procedure, data collection instruments, and intervention content were feasible and acceptable. The preparation study was conducted for 4 weeks. Four older adults were recruited through Purposive Sampling with two females and two males (Mean age = 69.3 years, SD = 3.30) (64).

The participants were asked to finish all the measurements once and to attend a 4-week blended intervention program. The intervention content was the same as the first 4-week out of the proposed 10-week intervention content in the main study. After finishing the pilot study, the participants were invited to give suggestions and feedback. Depending on the input from the participants (e.g., making web pages more accessible to operate, shortening the health talk), the intervention content was further adapted. The participants in the pilot study will not be involved in the main study.

### 2.6 Retention strategies

Different strategies will be applied to improve the adherence rate of the three assessments and the participation rate of all intervention sessions throughout the program. Such methods will include (1) a WhatsApp message will be sent to older adults in all groups 1 day before the interventions start to remind participants to participate by then; (2) there will be a check-in before each face-to-face class to help participants and researchers keep track of the attendance rate of all study participants throughout the course; (3) the printed materials of the face-to-face health talk will be distributed to the older adults in each face-to-face session. Since older people tend to have declining memories, this will help them look it up at home after they finish the course; (4) starting from the fifth week, the participants who document a valid diary three times a week on the website for each behavior will receive a cash reward of $10HKD, which will be distributed once a week; (5) after the completion of each data collection (T1, T2, and T3), the individualized health report including physical and mental health outcomes will be provided to the older adults.

### 2.7 Data analysis

All data will be analyzed by using SPSS 29.0. The Expectation Maximization (EM) method will be applied for missing values. Intention-to-treat principles will be used for all analyses. Independent samples *t*-tests, *F*-tests, and Chi-square tests will be used to compare the baseline characteristics at T1. Statistical significance will be set at the 5% level (two-tailed).

A series of generalized linear mixed models (GLMM) will be applied to evaluate the intervention effects on behaviors (PA, FVI, and MFEA), social-cognitive variables of behaviors (i.e., self-efficacy, planning, social support, and action control), physical health outcomes (i.e., physical fitness test, clinical indicators), mental health outcomes (i.e., depression, loneliness), and health-related quality of life (HRQoL).

### 2.8 Time schedule of the study

**Figure 3:**
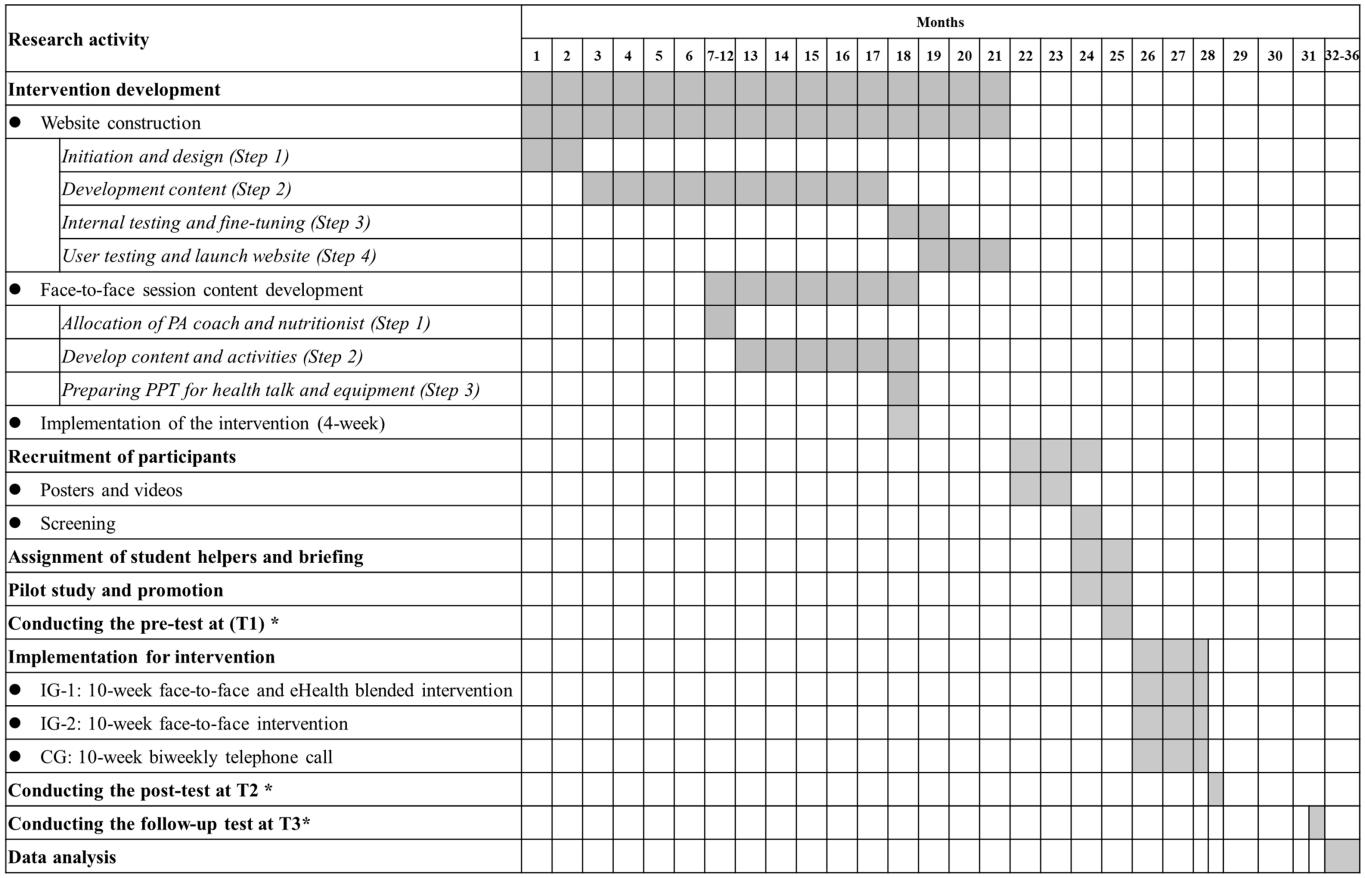
Gantt chart of research activities. *: The lengths of outcome tests at each time point, interventions, and follow-ups will be 1 month, 2.5 months (10 weeks), and 3 months, respectively.

The proposed study will be completed over 36 months, as shown in Figure 3.

## 3 Discussion

The high prevalence of inadequate PA and unhealthy dietary habits in the old population has garnered significant attention from health researchers and policymakers. Current research would investigate the effectiveness of a blended approach comprising face-to-face and eHealth interventions, specifically focusing on PA, dietary patterns, and health outcomes among older adults. It will be one of the first studies to apply blended intervention among older adults to promote healthy lifestyle behaviors theory- and evidence-based. Three unique aspects characterize the project.

The first unique aspect is the intervention channels, which include face-to-face workshops and eHealth sessions. The eHealth session will supplement traditional face-to-face treatment or seminars for older adults (65). Although the blended interventions are still under-explored, some potential benefits exist: The participants can review the intervention content anytime after the physical session. It is easier to get timely feedback from health care providers (e.g., doctors, health instructors, social workers), reducing the travel time, appointment process, and waiting time for feedback. It also helps increase the social interaction between healthcare providers (e.g., doctors, health instructors, social workers) and participants, reducing participants’ isolation after face-to-face contact. Besides, the healthcare providers can track the process and enhance adherence to the intervention (66).

The second unique aspect is that this is the first study applying eHealth technology to promote PA and diet behavior among Hong Kong community-dwelling older adults. Driven by the development of technology, more than 73.0% of Hong Kong’s older adults (≥ 65 years) have smartphones, and more than 70% use the Internet regularly in 2021 (67). Although many older adults have access to the Internet in Hong Kong, it is still difficult for them to use smartphones due to unfamiliarity with functions and unfriendly application design and operation (68). Hence, the stand-alone eHealth intervention might be complex for older people to adhere to. eHealth might be a complementary technology for the traditional face-to-face intervention project. It was found that 75% of people aged 65 years or above are suffering from one or more chronic diseases in Hong Kong. As these chronic diseases affect functionality and well-being, the old population accounts for more than 46% of patients’ hospital days (69). With the aging population and high prevalence of suffering from chronic diseases, this should be addressed immediately and appropriately to cope with the financial burden of treatment, rehabilitation, and residential care services. This study can develop and prove a blended intervention strategy for promoting lifestyle behaviors. It will be expected to be applied for disease management after the physical clinical.

The third unique aspect lies in exploring the psychosocial factors of behavior change and comprehensive health outcomes (BMI, physical fitness, clinical indicators, depression, loneliness, and health-related quality of life). The findings of this study can provide evidence about whether sufficient PA and a healthy diet are proven to be a protective factor for NCDs and associated with improvement in physical health, mental health, social well-being, cognitive function, and different aspects of body functions, which were proved by previous studies among other populations (70, 71). Besides, exploring the relationship between behavior change and psychosocial factors can enrich understanding mechanisms and have implications for theory-building.

Suggestions for further research encompass various aspects, including comparisons of a blended intervention with stand-alone eHealth interventions among older adults exploring aging effects and examining health lifestyle intervention effects in diverse geographical regions or countries. New research approaches are needed to enhance lifestyle behaviors, which can alleviate the growing demand for healthcare systems due to the aging population across the global landscape and within all medical and public health domains. Yet, identifying the critical predictive variables that underlie specific behavioral changes remains of utmost importance, a facet requiring comprehensive investigation in future research.

## Ethics statement

The studies involving humans were approved by the Research Ethics Committee at Hong Kong Baptist University. The studies were conducted in accordance with the local legislation and institutional requirements. The participants provided their written informed consent to participate in this study.

## Author contributions

MY: Conceptualization, Methodology, Project administration, Writing – original draft. YD: Conceptualization, Methodology, Project administration, Supervision, Writing – review & editing. SL: Conceptualization, Supervision, Writing – review & editing, Methodology. WL: Investigation, Supervision, Writing – review & editing. NS: Investigation, Writing – review & editing.
